# Artificial intelligence in nursing: Priorities and opportunities from an international invitational think-tank of the Nursing and Artificial Intelligence Leadership Collaborative

**DOI:** 10.1111/jan.14855

**Published:** 2021-05-18

**Authors:** Charlene Esteban Ronquillo, Laura-Maria Peltonen, Lisiane Pruinelli, Charlene H. Chu, Suzanne Bakken, Ana Beduschi, Kenrick Cato, Nicholas Hardiker, Alain Junger, Martin Michalowski, Rune Nyrup, Samira Rahimi, Donald Nigel Reed, Tapio Salakoski, Sanna Salanterä, Nancy Walton, Patrick Weber, Thomas Wiegand, Maxim Topaz

**Affiliations:** 1 Daphne Cockwell School of Nursing, Faculty of Community Services, Ryerson University, Toronto, ON, Canada; 2 School of Nursing, Faculty of Health and Social Development, University of British Columbia Okanagan, Kelowna, BC, Canada; 3 International Medical Informatics Association, Student and Emerging Professionals Special Interest Group; 4 Department of Nursing Science, University of Turku, Turku, Finland; 5 School of Nursing, University of Minnesota, Minneapolis, MN, USA; 6 Lawrence S. Bloomberg Faculty of Nursing, University of Toronto, Toronto, ON, Canada; 7 School of Nursing, Department of Biomedical Informatics, Data Science Institute, Columbia University, New York, NY, USA; 8 Precision in Symptom Self-Management (PriSSM) Center, Reducing Health Disparities Through Informatics Training Program (RHeaDI), Columbia University, New York, NY, USA; 9 Law School, University of Exeter, Exeter, UK; 10 School of Human & Health Sciences, University of Huddersfield, Huddersfield, UK; 11 Nursing Direction, Nursing Information System Unit, Centre Hospitalier Universitaire Vaudois (CHUV) Lausanne, Lausanne, Switzerland; 12 Leverhulme Centre for the Future of Intelligence, University of Cambridge, Cambridge, UK; 13 Department of Family Medicine, McGill University, Lady Davis Institute for Medical Research of Jewish General Hospital, Mila Quebec Artificial Intelligence Institute, Montreal, QC, Canada; 14 College of Medicine and Health, University of Exeter, Exeter, UK; 15 Department of Mathematics and Statistics, University of Turku, Turku, Finland; 16 Department of Nursing Science, University of Turku and Turku University Hospital, Turku, Finland; 17 Research Ethics Board, Women's College Hospital, Toronto, ON, Canada; 18 Health Canada and Public Health Agency of Canada's Research Ethics Board, Toronto, ON, Canada; 19 NICE Computing SA, Lausanne, Switzerland; 20 European Federation for Medical Informatics (EFMI); 21 ITU/WHO Focus Group on Artificial Intelligence for Health (FG-AI4H); 22 Fraunhofer Heinrich Hertz Institute, Berlin, Germany; 23 Berlin Institute of Technology, Berlin, Germany

**Keywords:** health services research, information technology, leadership, management, nurse roles, policy, politics, technology, workforce issues

## Abstract

**Aim:**

To develop a consensus paper on the central points of an international invitational think-tank on nursing and artificial intelligence (AI).

**Methods:**

We established the Nursing and Artificial Intelligence Leadership (NAIL) Collaborative, comprising interdisciplinary experts in AI development, biomedical ethics, AI in primary care, AI legal aspects, philosophy of AI in health, nursing practice, implementation science, leaders in health informatics practice and international health informatics groups, a representative of patients and the public, and the Chair of the ITU/WHO Focus Group on Artificial Intelligence for Health. The NAIL Collaborative convened at a 3-day invitational think tank in autumn 2019. Activities included a pre-event survey, expert presentations and working sessions to identify priority areas for action, opportunities and recommendations to address these. In this paper, we summarize the key discussion points and notes from the aforementioned activities.

**Implications for nursing:**

Nursing's limited current engagement with discourses on AI and health posts a risk that the profession is not part of the conversations that have potentially significant impacts on nursing practice.

**Conclusion:**

There are numerous gaps and a timely need for the nursing profession to be among the leaders and drivers of conversations around AI in health systems.

**Impact:**

We outline crucial gaps where focused effort is required for nursing to take a leadership role in shaping AI use in health systems. Three priorities were identified that need to be addressed in the near future: (a) Nurses must understand the relationship between the data they collect and AI technologies they use; (b) Nurses need to be meaningfully involved in all stages of AI: from development to implementation; and (c) There is a substantial untapped and an unexplored potential for nursing to contribute to the development of AI technologies for global health and humanitarian efforts.

## 1 Introduction

Artificial intelligence (AI) is defined as ‘… the science and engineering of making intelligent machines, especially intelligent computer programs’ (McCarthy, 1956). Increasingly sophisticated AI such as personalized advertisement and self-driving cars are revolutionizing a diverse range of professional sectors. In healthcare, AI is being adopted to aid healthcare professionals deliver high-quality care more efficiently and equitably. For example, AI can support less experienced healthcare professionals who may have fewer resources to still deliver high-quality care through learning from other’s experiences (e.g. identification of rare disease symptoms through massive database searches) (Schaefer et al., 2020).

In the context of nursing, examples of applications of AI demonstrate the potential impact that the use of these technologies can have in nursing practice. For example, speech recognition technologies can speed up and enhance nursing documentation (Fratzke et al., 2014; Monica, 2018) and machine learning has been used to develop a tool to aid nurses in using standardized technologies, by automatically suggesting the most relevant terms to be used based on the text written by the nurse (Moen et al., 2020). Other applications include text mining where AI technologies are being used to mine millions of nursing notes to identify patients with fall history (Topaz, Murga, Gaddis, et al., 2019) or drug and alcohol use disorders (Topaz, Murga, Bar-Bachar, et al., 2019), to support care planning and patient risk detection. Similarly, machine learning, specifically deep learning, has been experimented to predict pain sensation and physical deterioration for acute critical conditions (Pruinelli et al., 2018; Pruinelli, Stai, et al., 2019; Pruinelli, Westra, et al., 2019). In the near future, AI technology will be able to help nurses provide precise and individualized evidence-based care that meets patients’ goals and priorities. AI technologies will also help nurses integrate different types of relevant data (e.g. environmental, genomic, health data, socio-demographics) strengthening nurses’ capacity to provide multifaceted care. Moreover, a recent scoping review has highlighted that much of the research on AI in healthcare has focused on secondary and tertiary care, leaving still considerable opportunity to explore nurses’ use of AI in primary care (Abbasgholizadeh-Rahimi et al., 2020). From these examples, it is clear that nurses are not exempt from the proliferation of AI in healthcare systems, with AI often touted as tools that can transform the provision of health care and improve health outcomes (Clancy, 2020).

### 1. 1 Background

The dynamics between AI and nursing has yet to be critically interrogated. This is despite nurses being the largest group of healthcare professionals internationally (International Council of Nurses, 2017), and, by sheer volume of the workforce, nurses likely being the healthcare professionals who are most exposed to new AI technologies. Recognizing the mixed and complex, albeit limited, perspectives about AI in nursing, the Students and Emerging Professionals Special Interest Group in the International Medical Informatics Association organized the first international invitational expert think-tank workshop of the Nursing and Artificial Intelligence Leadership Collaborative (NAIL), titled ‘Artificial intelligence in nursing: social, ethical and legal implications’. The 3-day think-tank (October 23–25, 2019) held at the Brocher Foundation in Switzerland invited 19 interdisciplinary participants from Canada, Finland, Switzerland, the United States and the United Kingdom. The NAIL Collaborative comprises experts in AI development, AI implementation, nursing, and biomedical ethics, AI in primary health care, AI legal aspects, philosophy of AI in health, nursing practice, implementation science, high-level policymakers for healthcare institutions and international informatics groups, a representative of patients and the public, and Chair of the ITU/WHO Focus Group on Artificial Intelligence for Health. Activities included a pre-event survey to elicit attendees’ initial perspectives of AI in nursing, presentations by all invited attendees on their areas of expertise as related to AI and/or nursing and working sessions with attendees, to delve into in-depth discussions.

## 2 The Artificial Intelligence for Nursing: Ethical, Legal and Social Implications Invitational Think-Tank

### 2.1 Aims

In this paper, we summarize and highlight poignant points of discussion from the think-tank. These include central issues, priorities and key insights associated with AI technologies in nursing in the context of current discourses. We conclude the paper with actionable recommendations on issues related to the safe development, implementation and adoption of AI in nursing including the ethical, legal and social implications of AI technology.

### 2.2 Current discourse about AI’s impact on nursing

In nursing, advancements in AI technologies are often received with cautious excitement (Erikson & Salzmann-Erikson, 2016; Robert, 2019; Skiba, 2017). On the one hand, the use of AI presents the potential for optimizing nursing care delivery by alleviating mundane and time-consuming and burdensome tasks that do not require specialized nursing skills or knowledge (e.g. managing hospital room logistics, calling housekeeping for cleaning and restocking room supplies) and freeing up time for nurses to spend on direct (versus indirect) patient care. On the other hand, the use of AI concurrently introduces the risk for unintended consequences that can have a potential negative impact on the nursing profession.

AI technologies have the potential to propel nursing capabilities and enable nurses to provide more evidence-based and personalized care to their patients. AI technologies have the potential to support responsive and evidence-based nursing practice through the provision of cognitive insights and decision support, for example, through visualization of patient trends that can provide insights for both immediate patient care as well as long-term planning and management. Proponents of AI also point to the potential for AI to free-up time for healthcare professionals to dedicate in improving the relationships with patients (Topol, 2019). Indeed, the time that can be freed up for nurses can be spent on fostering relational care, supporting nurses’ ability to develop broader insights into the contexts of patients’ health. Moreover, time that is freed up for nurses can be spent on engaging with recent research and supporting up-to-date knowledge of the evidence to support practice, activities that are among the most common to be put aside for lack of time and opportunity (Duncombe, 2018). Better relationships with patients and up- to-date knowledge of the evidence, taken together, support nurses’ ability to provide personalized care that considers a holistic view of patients.

Along with the potential or positive outcomes, AI technologies can have unintended consequences that can have a potential negative impact on the nursing profession and on the main aims of nursing practice. For example, there exists the risk for AI to perpetuate or systematically embed existing human biases into systems (Benjamin, 2019), such as a recent case where a clinical decision algorithm introduced racial bias by prioritizing care for less sick white patients over sicker Black patients in the United States (Obermeyer et al., 2019). Beyond impacts on clinical and health outcomes, AI in nursing could also exacerbate the push towards market-driven goals of efficiency. There exists a very real potential to instead reallocate newly freed-up time towards increasing the volume of patients and tasks assigned to nurses. Hence efficiency goals (i.e. quantity of care) run the risk of eclipsing the opportunities that the use of AI in health systems are meant to create (i.e. quality of care).

Such negative impacts are not inevitable. For instance, AI also has the potential to make visible and remove human bias and improve decision making (Leibert, 2018), for example by discovering and quantifying the impact of taken for granted variables such as sex, gender, ethnicity, or race (while we recognize that race has no scientific meaning, experiences of racism have clear links to health outcomes), for which our understanding of impacts are emergent (Davenport & Kalakota, 2019). Ensuring the best possible consequences from AI for nursing will depend on which values and priorities end up guiding the development of AI tools, and whether they implemented with an adequate understanding of both their potentials and limitations.

Placed in nurses’ hands, unintended consequences of using AI tools can be direct and serious, reflecting the same concerns discussed by O’Keefe-Mccarthy (2009) in their classical discussion of the mediating role of technology in the nurse-patient encounter and the subsequent effects on the moral agency of nurses. Given the potential magnitude of the impact of AI tools, there is an ethical imperative for nurses to have a minimum basic understanding of how these tools come to be developed, what informs them, and the implications of using such tools on their clinical judgement and practice. The responsibility of having a minimum understanding of AI that all nurses must develop is arguably no different from the requirement of nurses to have a basic understanding and competency in the use of any type of new technology or tool that they use in their practice.

Notwithstanding these important implications of AI for the nursing profession, there is a growing, but still a limited critical discourse in the nursing literature (Brennan & Bakken, 2015; Linnen et al., 2019). In the sphere of nursing education, addressing AI remains, largely, absent. Nursing curricula continue to struggle with incorporating basic nursing informatics competencies as part of basic nursing education (Ronquillo et al., 2017; Topaz et al., 2016), which will become more worrisome given the growing interest in using AI tools in health systems. In other words, there is the potential that the challenges that nurses currently face regarding the effective use of and potential for leading innovations in health information technologies can be further compounded if a gap in AI knowledge is added to existing gaps in basic health informatics knowledge.

## 3 Discussion

### 3.1 Away forward for AI in nursing

The following represent a summary of the discussion points identified in the NAIL Collaborative think-tank discussions, framed as pressing priorities for the nursing profession. Each priority point is introduced with the identification of a current gap in understanding or use of AI in relation to nursing practice. For each identified gap, we propose strategies and opportunities--with implications for nursing practice, education, research and leadership—that can be pursued to ensure the appropriate and safe use of AI in nursing and enable the nursing profession to use AI tools to optimize health outcomes.

### 3.2 Priority 1. Nurses must understand the relationship between data they collect and AI technology that they use

Gap: Nurses are the group of healthcare professionals who generate the most data in health systems, as they complete the most documentation (Collins et al., 2018). Nurses play an important role in collecting data that might be eventually used by AI tools, as evidenced by work that has linked the nature and patterns of nursing documentation practices with patients’ mortality (Collins et al., 2013). There nevertheless appears to be limited understanding of the link between nursing documentation and how these documents may be used for purposes beyond immediate clinical decision making, administrative reporting and keeping a legal record as taught in basic nursing education. While understanding these aspects of documentation has been sufficient to inform nursing practice in the past, we argue that nurses should also understand the relationship between their clinical documentation and AI. For one, understanding the nature and quality of data that are collected and documented as part of the nursing practice, can and do, directly inform AI tools. Also, AI-based clinical decision support has various levels of uncertainty that requires clinician interpretation (Shortliffe & Sepulveda, 2018). When deciding to follow an AI-based recommendation, nurses serve as the last line of evaluation for the appropriateness of an intervention (Eisenhauer et al., 2007). Moreover, a significant current challenge is that many nursing educational programmes—both in entry-level nursing education and continuing education of professional nurses—do not have enough expertise in teaching health informatics and AI technologies (Cummins et al., 2016; Mantas & Hasman, 2017) to effectively address this gap in AI understanding.

### 3.3 Strategies and opportunities to address priority 1

To bridge the educational gap, there is a need to develop a curriculum with ‘minimum AI in nursing competencies’, a set of domains and concepts that all entry-level nurses should receive as part of their basic nursing education (Michalowski, 2019). Some organizations, such as the American Association of Colleges of Nursing (AACN), are moving to a competency-based education with a technology domain crossing over all domains due to the current need for this topic in all levels of nursing education. Similar efforts concurrently need to be made to support the development of these competencies among practising nurses, as well as nurse leaders (Pruinelli et al., 2020), where this material can be delivered through continuing education initiatives. Graduate nursing education also would benefit from the creation of opportunities for advanced AI education as well as the formation of sub-specializations in AI under health informatics programs. Specific recommendations are outlined in the summary Table 1 towards ensuring that a curriculum with ‘minimum AI in nursing competencies’ can be met, with the goal of having all nurses hold basic knowledge and competence related to AI use in nursing.

### 3.4 Priority 2. Nurses must be involved in all stages of AI: From development to implementation

Gap: Currently, nurses are often end-users of technologies that incorporate AI (e.g. advanced clinical decision support) rather than collaborators in development. As such, there are other calls for nursing: to take the driver’s seat in determining which aspects of nursing care can be delegated and to be key actors in introducing AI technologies in health systems (Pepito & Locsin, 2019). In a clinical context, the AI development lifecycle must start with a thorough understanding of the clinical question and clinical work-flows, as these ultimately shape the successful use and subsequent impact of these technologies on patient and organizational outcomes. AI development teams should be interdisciplinary, including nurses, to ensure that contributions of computer science and engineering members of teams are grounded in clinical realities of the provision of patient care.

Nurses’ contributions to all stages of the AI development lifecycle become crucial when recognizing the intertwining of the consequences accompanying the use of AI in nursing (both positive and negative) with the foundational underpinning of the nursing profession as being concerned with beneficence towards patients, communities, and populations, and advocacy for social justice (Paquin, 2011; Stievano & Tschudin, 2019; Wilmot, 2012). Patient, family, and community advocacy and the promotion of person-centred care comprise foundational functions of the nurse. As such, nurses are uniquely positioned to propose how the impact of AI should be measured in terms of nursing and patient outcomes. It is through active participation in all aspects of the AI development lifecycle (Matinolli et al., 2019) that unique insights from nursing can contribute to the thoughtful development and use of AI that optimize potential benefits and minimize potential negative consequences for patients, communities, populations, healthcare systems and the nursing profession.

### 3.5 Strategies and opportunities to address priority 2

Nurses need to be meaningfully (rather than tokenistically) involved and contribute as key members of AI development and implementation teams in health systems. While nursing can contribute in many ways across the AI development lifecycle, we have identified three potential distinct and important informant/communicator roles that can be contributed by nursing. These include: (a) delineating clinical problems; (b) serving as intermediaries between the clinical and technical spheres; and (c) incorporating features of relational practice (Dykes & Chu, 2020). Nurses’ expertise and deep familiarity with working closely with patients should be tapped into, to better delineate clinical problems that AI technologies aim to address. For example, when predictive algorithms are being developed from clinical data, nurses can contribute with practice-based perspectives to technical teams (often consisting of engineers, computer scientists, user interface design experts, etc.) to understand why some data elements are missing or incomplete (e.g. poor documentation of social risk factors) (Navathe et al., 2018) and offer potential strategies to address these shortcomings. Closely related is the potential for nurses to serve as key intermediaries between technical experts developing solutions and nurses as clinical end-users (Dykes & Chu, 2020). These two groups speak very different professional languages and nurses educated in AI concepts are perfect for bridging this vocabulary gap. Finally, nursing expertise in relational practice (i.e. understanding and focus on the quality of human relationships) represents a unique strength to contribute to the AI development lifecycle. The primacy of nurse–patient relationship as a defining priority of nursing can contribute greatly to AI applications in robotics and elsewhere. Nurses can provide insight into the value of empathy and human touch, the role these concepts play in therapeutic relationships (Dobson et al., 2002; Kerr et al., 2019), and the dynamics between AI technologies and human relationships that need to be considered throughout the AI development lifecycle.

### 3.6 Priority 3. ‘AI for Good Nursing’ (AI4GN)

Gap: There is a limited recognition of the relationship between AI technologies and the nursing profession as related to the contribution towards global (and oftentimes national) health and humanitarian efforts. There are numerous movements focused on the use of ‘AI for good’ in the academic, non-profit and industry spheres (e.g. Google’s AI for Social Good, Google AI, 2018; Microsoft’s AI for Good, Microsoft, 2020; AI for Good Foundation, AI for Good Foundation, 2015), AI for Good Global Summit (International Telecommunications Union, 2020), advocating for the use of AI to benefit humanity and address difficult social, economic, environmental, health and humanitarian challenges around the globe. Despite the potentially significant impact of AI technologies on nursing work, there remain to be efforts from nursing relating to the notion of using AI4GN, or the use of AI technologies in nursing to achieve a greater good for the profession and for populations.

### 3.7 Strategies and opportunities to address priority 3

Efforts that recognize the contributions that fall in AI4GN can include leveraging the unique positionality of nurses in healthcare systems towards advocating for the inclusion of equity and social justice considerations in the development and implementation of AI technologies in health systems. Nurses are health professionals who spend the most time with patients and are often referred to as the most trusted profession (Colduvell-Gaines, 2019). Nurses are well situated to identify potential biases in data collection (e.g. decontextualized data that does not consider the impact of systemic structures on individuals) which can lead to the embedding of these biases in the AI tools developed. As well, nurses are ideally situated to identify ethical concerns relating to the implementation and use of AI tools (e.g. highlighting the problematic nature of using facial recognition tools) and instances that can exacerbate existing inequities and cause potential harm among particular groups and populations. For example, a recent study highlights the greater likelihood of digital data being collected and shared from children’s use of apps when those children come from lower-education backgrounds (Zhao et al., 2020). In the context of the healthcare system, this translates to an important facet of nursing education that needs to be developed and embedded as a routine component of a holistic nursing assessment and intervention. Namely, this comprises educating patients and families about digital literacy, digital privacy, laws and regulations on data collection and protection of digital health data and how these all relate to the AI tools that are used in healthcare provision.

## 4 Conclusion

AI technologies will change the profession of nursing. AI technologies can serve as important tools to support the contribution of nurses towards higher level aims of evolving the nursing profession and improving population and global health.

If nursing takes a proactive role in addressing these above-mentioned priorities, AI has the potential to enhance and extend nursing capabilities. In return, nursing has much to contribute to the development of AI systems that leverage nurses’ strengths and expertise in relational practice and patient advocacy, towards the development of AI that considers patients with a more holistic view. It is important to note that all priority areas discussed in this paper are necessarily linked. They do not each sit on their own but inform a broad purposeful approach to empowering nurses in their active involvement in all aspects of AI in health care. We argue that nurses have a responsibility to know about the AI technology they use, as has been stated from an industry perspective (McGrow, 2019). Moreover, there is a great opportunity for AI tools to support nurses’ problem-solving abilities and identify solutions for optimizing care provision (Cato et al., 2020). There is nevertheless a need for support from health systems stakeholders and high-level decision-makers to facilitate the ability of the nursing profession to address these identified priorities. The priorities presented in the paper are summarized in Table 1, alongside a list of specific recommendations based on the strategies and opportunities outlined in this paper.

## Figures and Tables

**Table 1:** Actionable suggestions based on the identified priority areas.

Priority	Recommendations			
	Education	Practice	Research	Leadership
1.Nurses must understand the relationship between data they collect and AI technology	Nursing educators should consider the creation of a regional AI4N Taskforce to develop the ‘Minimum AI in Nursing Competencies’ curriculum for nursing undergraduate education (linked with Priority 3). Nursing educational programmes and continuing education should prioritize recruiting faculty with expertise in health informatics and technology development. Nursing educational programmes that are unable to recruit faculty with health informatics or technology development expertise have a responsibility to identify alternative ways of ensuring content related to the ‘minimum AI in nursing competencies’ are delivered. This can be achieved by partnering with professional health informatics groups and/or experts at other institutions and/or partnering with computer or information science departments. Nursing educators need to ensure that nursing curricula at all levels should have appropriate integration of AI knowledge to ensure nurses are equipped to practice with the knowledge, skills and judgement required to work in health systems that use Al.	Nursing leaders should create an organizational AI4N Taskforce to develop the ‘Minimum AI in Nursing Competencies’ curriculum for practising nurses (linked with Priority 3) that can be delivered as part of new employee orientations and as continuing education. Nurses need options for in-practice training on the specific AI technologies they use. Nursing stakeholders need to create structures that promote a continuous discussion of the implications of AI technologies in nursing on all levels. Nursing organizations need to develop guidelines for the implementation of AI technologies to ensure safe use of AI. AI-system developers need to make AI-system outputs transparent for nurses.	Nursing researchers should focus on the use and impact of AI in nursing and the impacts related to workforce, clinical and patient health outcomes as well as making the AI lifecycle explainable, from AI conception to implementation. Nursing researchers should focus on the contributions of nursing to AI technology development and implementation.	Nursing leaders need to have an understanding of AI technologies to be able to lead the implementation of these technologies and support clinical teams on its use. Nursing leaders need to create opportunities for further education and training on AI4N for staff (educators and clinicians). Nursing leaders need to promote nurses’ attitudes towards learning about the AI technologies they use.
2. Nurses must be involved in all stages of AI creation: from development to implementation	Educational institutions should facilitate the development of partnerships and collaborations between nursing educators and technology teams, to provide nursing students in all levels an opportunity to work in an interdisciplinary setting and get involved in technology development. Existing examples of such programmes can be used to inform the development of bespoke programmes (e.g. see the University of Turku’s Master’s joint degree programme in future health and technology that accepts both nursing students and technology students; University of Turku, 2020). Nursing educators should acknowledge current theories on technology development to support the rigour and the respect to all stages of technology development for secure and safe AI products. Nursing educators should develop advanced educational training for nurses who are interested in taking on more active and hands-on roles in the development and implementation of AI technologies in health systems.	Nurses should play an active role in AI technology development and deployment in clinical settings to ensure that technology is integrated into the clinical workflows, patient and caregiver perspectives are addressed and potential unintended consequences are forecasted.	Research entities and funding mechanisms should encourage participatory and co-produced research designs in health AI research to leverage nursing expertise in relational practice. Research entities and funding mechanisms needed to support the development of AI or related technologies that target nursing practice and establish programmes of research in this underdeveloped field.	Leaders should build organizational structures to afford nurses opportunities to be involved in all stages of AI creation.
3. “AI for good nursing”: AI must be used to help nurses be better at what they do	Nursing education programmes can use virtual environments and/or simulations mirroring real case studies to study AI implications. These would focus on the provision of patient-centred and relational care while using AI technologies; assessment of patients’ digital literacy and digital privacy and security as part of the informed consent process; understanding the impacts of AI technology use on practice. Nursing educators need to leverage data already collected (e.g. simulation labs) to further develop nursing education and support critical thinking.	All stakeholders need to ensure that AI technology should be used to help nurses allocate more time for providing preventative health recommendations to patients and patient populations. All stakeholders need to ensure that AI technologies (e.g. clinical decisions support systems) incorporate a holistic patient perspective, support care provision based on patient’s goals and priorities, and proactively consider ethical concerns that can arise from using the technology, as part of the development process. All stakeholders need to ensure that AI technology supports fundamental care processes in a way that supports critical thinking and meaningful care decisions. Practising nurses need to ensure they are knowledgeable about potential areas of bias related to data collection and subsequent use in AI technologies (e.g. identification of decontextualized data, identification of potential areas where existing inequities may be exacerbated by AI tools). Al-developers need to ensure that clear guidance, protocols, and systems need to be developed and established in healthcare organizations to enable nurses to flag AI technologies being used that are potentially questionable, result in patient harm, or exacerbate existing inequities.	Nursing researchers need to study what types of AI technologies are needed to augment nursing critical-thinking and care skills. Nursing researchers need to examine how AI is going to impact nursing workflow and care outcomes. Nursing researchers need to explore how equity and social justice considerations can be incorporated in the design and development of AI technologies.	Health systems leaders and nursing leadership need to ensure that achieving economic efficiencies is not the sole driver of AI implementation; AI technologies can be used to help nurses with specific skill-based tasks to afford more time for higher-order cognitive tasks and critical thinking. There are existing efforts that can be built on to better evaluate the impacts of AI technologies on quality of care. For example, the work towards developing metrics of nursing value from electronic health records (Pruinelli et al., 2016; Welton & Harper, 2016). Nurse leaders should be key advocates to ensuring that AI use takes a more proactive, rather than a reactive approach that is currently seen in healthcare. This includes ensuring that key variables for nursing care and outcomes, and variables related to social determinants of health and equity are considered in predictive modelling and development of clinical decision support systems. Leaders should also be key proponents for data integration and the combination of multiple data sources to provide more valuable insights than those available in single sources. Leaders should also be proactive in identifying opportunities for massive data where the biggest potential lurks, based on understandings of nursing practice and subsequent impacts on populations.

## Data Availability

The data that support the findings of this study are available from the corresponding author on reasonable request.

## References

[R1] Abbasgholizadeh-Rahimi S, Granikov V, Pluye P (2020). Current works and future directions on application of machine learning in primary care.

[R2] AI for Good Foundation (2015). AI for good foundation.

[R3] Benjamin R (2019). Race after technology: Abolitionist tools for the new Jim code. Social Forces.

[R4] Brennan PF, Bakken S (2015). Nursing needs big data and big data needs nursing. Journal of Nursing Scholarship.

[R5] Cato KD, McGrow K, Rossetti SC (2020). Transforming clinical data into wisdom: Artificial intelligence implications for nurse leaders. Nursing Management.

[R6] Clancy TR (2020). Artificial intelligence and nursing: The future is now. JONA.

[R7] Colduvell-Gaines K (2019). Nurses rank most honest profession 17 years in a row.

[R8] Collins SA, Cato K, Albers D, Scott K, Stetson PD, Bakken S, Vawdrey DK (2013). Relationship between nursing documentation and patients’ mortality. American Journal of Critical Care.

[R9] Collins S, Couture B, Kang MJ, Dykes P, Schnock K, Knaplund C, Chang F, Cato K (2018). Quantifying and visualizing nursing flowsheet documentation burden in acute and critical care. AMIA.

[R10] Cummins MR, Gundlapalli AV, Gundlapalli AV, Murray P, Park H-A, Lehmann CU (2016). Nursing informatics certification worldwide: History, pathway, roles, and motivation. Yearbook of Medical Informatics.

[R11] Davenport T, Kalakota R (2019). The potential for artificial intelligence in healthcare. Future Healthcare Journal.

[R12] Dobson S, Upadhyaya S, Conyers I, Raghavan R (2002). Touch in the care of people with profound and complex needs: A review of the literature. Journal of Learning Disabilities.

[R13] Duncombe DC (2018). A multi-institutional study of the perceived barriers and facilitators to implementing evidence-based practice. Journal of Clinical Nursing.

[R14] Dykes S, Chu CH (2020). Now more than ever, nurses need to be involved in technology design: Lessons from the COVID-19 pandemic. Journal of Clinical Nursing.

[R15] Eisenhauer LA, Hurley AC, Dolan N (2007). Nurses’ reported thinking during medication administration. Journal of Nursing Scholarship.

[R16] Erikson H, Salzmann-Erikson M (2016). Future challenges of robotics and artificial intelligence in nursing: What can we learn from monsters in popular culture?. The Permanente Journal.

[R17] Fratzke J, Tucker S, Shedenhelm H, Arnold J, Belda T, Petera M (2014). Enhancing nursing practice by utilizing voice recognition for direct documentation. Journal of Nursing Administration.

[R18] Google AI (2018). AI for social good.

[R19] International Council of Nurses (2017). Who we are.

[R20] International Telecommunications Union (2020). AI for good global summit.

[R21] Kerr F, Wiechula R, Feo R, Schultz T, Kitson A (2019). Neurophysiology of human touch and eye gaze in therapeutic relationships and healing: A scoping review. JBI Database of Systematic Reviews and Implementation Reports.

[R22] Leibert F (2018). AI will improve healthcare and cut costs – If we get these 4 things right.

[R23] Linnen DT, Javed PS, D’Alfonso JN (2019). Ripe for disruption? Adopting nurse-led data science and artificial intelligence to predict and reduce hospital-acquired outcomes in the learning health system. Nursing Administration Quarterly.

[R24] Mantas J, Hasman A (2017). IMIA educational recommendations and nursing informatics. Studies in Health Technology and Informatics.

[R25] Matinolli HM, Mieronkoski R, Salanterä S (2019). Health and medical device development for fundamental care: Scoping review. Journal of Clinical Nursing.

[R26] McCarthy J (1956). What is artificial intelligence?.

[R27] McGrow K (2019). Artificial intelligence: Essentials for nursing. Nursing.

[R28] Michalowski M (2019).

[R29] Microsoft (2020). AI for good Microsoft.

[R30] Moen H, Hakala K, Peltonen L-M, Matinolli H-M, Suhonen H, Terho K, Danielsson-Ojala R, Valta M, Ginter F, Salakoski T, Salanterä S (2020). Assisting nurses in care documentation: From automated sentence classification to coherent document structures with subject headings. Journal of Biomedical Semantics.

[R31] Monica K (2018). Using EHR voice recognition to improve clinical documentation, usability.

[R32] Navathe AS, Zhong F, Lei VJ, Chang FY, Sordo M, Topaz M, Navathe SB, Rocha RA, Zhou L (2018). Hospital readmission and social risk factors identified from physician notes. Health Services Research.

[R33] Obermeyer Z, Powers B, Vogeli C, Mullainathan S (2019). Dissecting racial bias in an algorithm used to manage the health of populations. Science.

[R34] O’Keefe-Mccarthy S (2009). Technologically-mediated nursing care: The impact on moral agency. Nursing Ethics.

[R35] Paquin SO (2011). Social justice advocacy in nursing: What is it? How do we get there?. Creative Nursing.

[R36] Pepito JA, Locsin R (2019). Can nurses remain relevant in a technologically advanced future?. International Journal of Nursing Sciences.

[R37] Pruinelli L, Delaney CW, Garcia A, Caspers B, Westra BL (2016). Nursing management minimum data set: Cost-effective tool to demonstrate the value of nurse staffing in the big data science era. Nursing Economics.

[R38] Pruinelli L, Johnson SG, Fesenmaier B, Winden TJ, Coviak C, Delaney CW (2020). An applied healthcare data science roadmap for nursing leaders: A workshop development, conceptualization, and application. Computers, Informatics, Nursing.

[R39] Pruinelli L, Simon GJ, Monsen KA, Pruett T, Gross CR, Radosevich DM, Westra BL (2018). A holistic clustering methodology for liver transplantation survival. Nursing Research.

[R40] Pruinelli L, Stai B, Ma S, Pruett T, Simon GJ (2019). A likelihood-based convolution approach to estimate event occurrences in large longitudinal incomplete clinical data.

[R41] Pruinelli L, Westra BL, Westra T, Monsen KA, Gross C, Radosevich DR, Ma S, Simon GJ (2019). A multi-dimensional general health status concept to predict liver transplant mortality.

[R42] Robert N (2019). How artificial intelligence is changing nursing. Nursing Management.

[R43] Ronquillo C, Topaz M, Pruinelli L, Peltonen LM, Nibber R (2017). Competency recommendations for advancing nursing informatics in the next decade: International survey results. Studies in Health Technology and Informatics.

[R44] Schaefer RH, Lehne M, Schepers J, Prasser F, Thun S (2020). Theuse of machine learning in rare diseases: a scoping review. Orphanet Journal of Rare Diseases.

[R45] Shortliffe EH, Sepulveda MJ (2018). Clinical decision support in the era of artificial intelligence. JAMA.

[R46] Skiba DJ (2017). Augmented intelligence and nursing. Nursing Education Perspectives.

[R47] Stievano A, Tschudin V (2017). The ICN code of ethics for nurses: A time for revision. International Nursing Review.

[R48] Topaz M, Murga L, Bar-Bachar O, Cato K, Collins S (2009). Extracting alcohol and substance abuse status from clinical notes: The added value of nursing data. Studies in Health Technology and Informatics.

[R49] Topaz M, Murga L, Gaddis KM, McDonald MV, Bar-Bachar O, Goldberg Y, Bowles KH (2019). Mining fall-related information in clinical notes: Comparison of rule-based and novel word embedding-based machine learning approaches. Journal of Biomedical Informatics.

[R50] Topaz M, Ronquillo C, Peltonen LM, Pruinelli L, Sarmiento RF, Badger MK, Ali S, Lewis A, Georgsson M, Jeon E, Tayaben JL (2016). Advancing nursing informatics in the next decade: Recommendations from an international survey. Studies in Health Technology and Informatics.

[R51] Topol E (2019). The Topol review: preparing the healthcare workforce to deliver the digital future.

[R52] University of Turku (2020). Master’s degree programme in future health and technology.

[R53] Welton JM, Harper EM (2016). Measuring nursing value from the electronic health record. Studies in Health Technology and Informatics.

[R54] Wilmot S (2012). Social justice and the Canadian Nurses Association: Justifying equity. Nursing Philosophy.

[R55] Zhao F, Egelman S, Weeks HM, Kaciroti N, Miller AL, Radesky JS (2020). Data collection practices of mobile applications played by preschool-aged children. JAMA Pediatrics.

